# The Impact of Neoadjuvant versus Adjuvant Chemotherapy on Survival Outcomes in Locally Advanced Breast Cancer

**DOI:** 10.3390/curroncol31100448

**Published:** 2024-10-08

**Authors:** Farhad Ghasemi, Muriel Brackstone

**Affiliations:** Division of General Surgery, Western University, London, ON N6A 3K7, Canada; fghasemi2019@meds.uwo.ca

**Keywords:** locally-advanced breast cancer, delay, neoadjuvant, pre-operative chemotherapy

## Abstract

The utility of neoadjuvant chemotherapy is expanding in the treatment of breast cancer. Although individual trials have shown comparable survival between patients receiving neoadjuvant and adjuvant chemotherapy, large-scale data analyses for outcomes in patients with locally advanced breast cancer (LABC) are lacking. We conducted an individual-level statistical analysis using patients from six randomized controlled trials (RCTs) investigating survival outcomes with neoadjuvant versus adjuvant chemotherapy in breast cancer by abstracting and analyzing only the patients with LABC. Individual patient data for 779 patients with LABC were collected from six RCTs. Overall and disease-free survival rates were compared between patients receiving neoadjuvant vs. adjuvant chemotherapy with the Cox hazard model and log-rank statistics. Since chemotoxicity causing delays to surgical care is a potential drawback of neoadjuvant chemotherapy, local cohort data were then employed to assess the actual incidence of this, along with the causes behind any delays to surgery in patients receiving neoadjuvant chemotherapy. A time interval from neoadjuvant chemotherapy to surgery of >8 weeks was investigated in a local cohort of 563 patients, representing all locally treated patients receiving neoadjuvant chemotherapy between 2006 and 2019. The statistical analysis demonstrated no overall or disease-free survival differences in LABC patients receiving neoadjuvant vs. adjuvant chemotherapy (*p* = 0.96 and 0.74, respectively). Within our cohort, 31 (5.5%) patients treated with neoadjuvant chemotherapy experienced a delay of >8 weeks to surgery, with only 13 (2.3%) attributed to chemotherapy-related complications. Our study provides further support for the paradigm shift towards delivering chemotherapy for breast cancer patients in the neoadjuvant setting.

## 1. Introduction

The use of neoadjuvant chemotherapy in breast cancer is expanding. Initially used to downstage an inoperable breast cancer to make it surgically resectable, neoadjuvant chemotherapy offers additional advantages. These include providing an in vivo assessment of tumor response to the systemic treatment, enhancing breast conservation rates, and reducing the extent of axillary metastases prior to surgery [1,2]. 

Individual trials have demonstrated equivalent survival outcomes between patients receiving systemic therapy before or after surgery [3,4,5,6,7]. Epidemiologic data indicate that outcomes in locally advanced breast cancer (LABC) differ from those of early-stage disease, with higher rates of distant metastasis and worse survival outcomes [8]. While a large-scale meta-analysis of early-stage breast cancer patients receiving preoperative chemotherapy is available in the literature [9], such large-scale data are lacking for patients with LABC.

Timing between treatments is an important clinical consideration in the multimodal management of breast cancer [10]. Prolonged intervals between the completion of neoadjuvant chemotherapy and surgery have been linked with poorer outcomes [11]. In a study of breast cancer patients undergoing neoadjuvant chemotherapy, subgroup analysis showed a time interval of >8 weeks was associated with worse overall survival (HR 1.62, *p* = 0.02). This raises a potential concern for administering neoadjuvant chemotherapy, as medical complications arising from the treatment could delay surgery and, consequently, negatively impact oncologic outcomes. At the same time, several landmark trials have demonstrated a significant survival advantage with the addition of adjuvant-targeted therapies in patients who have residual disease following neoadjuvant chemotherapy [12,13]. This signifies the risk of undertreatment with upfront surgery.

In this study, we address the important question of survival outcomes for LABC patients receiving neoadjuvant chemotherapy through a combined statistical analysis of individual patient data from six randomized controlled trials. Due to the limited information on the timing of surgery and the reasons for prolonged intervals in available trial data, we then utilized local cohort data to address the concerns about delays to surgery and to quantify the incidence of chemotherapy-related complications causing any such delays. 

## 2. Materials and Methods

### 2.1. Identification of Studies and Patient Selection for the Combined Statistical Analysis

A PubMed search was carried out to identify RCTs comparing survival rates and relapse time between neoadjuvant and adjuvant therapy of breast cancer. Search criteria were set as follows: (breast cancer) AND (random* control trial) AND (neoadjuvant chemotherapy) AND (adjuvant chemotherapy) AND (locally advanced). The primary endpoints were overall survival (OS) and disease-free survival (DFS). Contacts were also made with international LABC expert oncologists. The referenced literature within all articles was further screened for appropriate RCTs. 

In all RCTs meeting the search criteria, clinical data (chemotherapy regimen, radiotherapy details, surgical procedures, locoregional recurrences, distant metastases) and survival data (treatment-related and cancer-related deaths, post-operative deaths due to complications) were extracted from the original publications. Each patient enrolled in the studies was assessed for the presence of LABC. 

### 2.2. Defining Patients with Locally Advanced Breast Cancer (LABC)

LABC was defined as a primary tumor size exceeding either 5 cm (T3) or 3 cm (T2), with greater than 3 positive nodes (N2 or N3); this was the case in five RCTs identified. In one RCT [7], tumor size and number of positive nodes were not recorded; instead, tumors were categorized by the ‘tumor, node, metastasis’ (TNM) system [14].

For the purposes of the combined statistical analysis, the following specific inclusion criteria were applied to each identified study to extract and merge data from all LABC patients:

- Wolmark et al. [15]: equal to or greater than 4 positive nodes, or clinical tumor size equal to or greater than 5 cm;
- Deo et al. [3]: all patients had LABC (T4a, N0-2, M0);
- Gazet et al. [4]: stage T3a/b, T4b/c;
- Makris et al. [5]: clinical tumor size equal to or greater than 5 cm, or clinical N stage equals 3;
- Mauriac et al. [6]: clinical tumor size equal to or greater than 5 cm;
- Van der Hage et al. [7]: pathological stage T3 or T4a/b/c/d.

### 2.3. Statistical Analysis Methodology of Combined Individual Patient Data

Permission to use the entire dataset from each of the original studies that met our inclusion criteria was obtained from each publication author. Rather than pooling trial data, individual-level data from patients with LABC in each RCT were abstracted to subsequently perform statistical analyses on this patient population from all six RCTs. Analysis was performed using SAS 9.2 software (SAS Institute Inc., Cary, NC, USA). The primary endpoints for the analysis were overall survival (OS) and disease-free survival (DFS) based on the data at the end of the treatment. The effect of the treatment type (neoadjuvant versus adjuvant) was assessed using the Wilcoxon test and log-rank statistics. 

For each measure of survival, the hazard ratio (HR) was calculated using the Cox proportional hazards model. Trial heterogeneity was evaluated using the interaction term between trial and intervention (neoadjuvant vs. adjuvant chemotherapy). Using the combined data for homogeneous trials, survival and the relapse-free interval were estimated using the Kaplan–Meier technique. Cox regression was used to compare the two treatment groups, with statistical adjustment for trial and other patient characteristics. A *p*-value threshold of <0.05 was set for statistical significance. 

### 2.4. Local Data Analysis on the Incidence and Cause of Delay to Surgery

The data from all patients treated at our institution with breast cancer were collected prospectively, including comprehensive patient demographics, pathology and treatment details, and life-long follow-up. A retrospective analysis of patients with breast cancer between 2006 and 2019 who received neoadjuvant chemotherapy was completed. Patients with metastatic breast cancer on presentation or those who received neoadjuvant endocrine therapy were excluded from the dataset. Breast cancer subtype was assigned based on estrogen receptor (ER), progesterone receptor (PR), Human epidermal growth factor receptor 2 (Her2) status, and tumor grade as per Supplementary Table S1. Early-stage breast cancer was defined as patients with tumors ≤5 cm and <4 nodes involved. 

Time to surgery was defined as the time interval between the last dose of cytotoxic chemotherapy and the surgical date. Delay to surgery was defined as the time to surgery of over 8 weeks (56 days) [11]. Further chart review was completed on the patients experiencing a time delay to surgery, and the reasons for the delay were grouped as follows: 1. administrative/non-medical reasons, 2. clinical trial protocol, 3. medical reasons unrelated to chemotherapy (ex. fracture due to mechanical fall or trauma), or 4. chemotoxicity. Delay due to chemotoxicity was defined as an adverse reaction to chemotherapy impeding timely surgical intervention. 

R statistical environment (version 4.3.1) was used for statistical analysis. Clinical data, including age, T stage, N stage, ER status, PR status, Her2 status, tumor grade, and subtype, were compared between patients with and without delay to surgery. Wilcox rank–sum test was used for age. Fisher’s exact test was used to compare ER status, PR status, and Her2 status between the groups, and Pearson’s chi-squared test was used for T stage, N stage, grade, and subtype. 

## 3. Results

### 3.1. Survival Outcomes in Patients with LABC Receiving Neoadjuvant Chemotherapy

Six RCTs [3,4,5,6,7,15] comparing survival rates and relapse time between neoadjuvant and adjuvant chemotherapy for breast cancer were identified through the search. A total of 2306 patients were included in these trials, 779 (33.8%) of which were identified as having LABC (Supplementary Table S2). 

Baseline characteristics, including mean age, hormone receptor status, surgery received, clinical nodal status, and N stage by pathology for LABC patients by trial, are summarized in Table 1 and Table 2. 

The HR for disease-free survival and overall survival between neoadjuvant versus adjuvant chemotherapy was found to not be significantly different when evaluated in each of the trials separately (Supplementary Table S3). Covariance matrices, compiled to test the homogeneity of data between studies, identified no significant deviations (DFS log-rank *p* = 0.716, OS log-rank *p* = 0.104).

**Figure 1:**
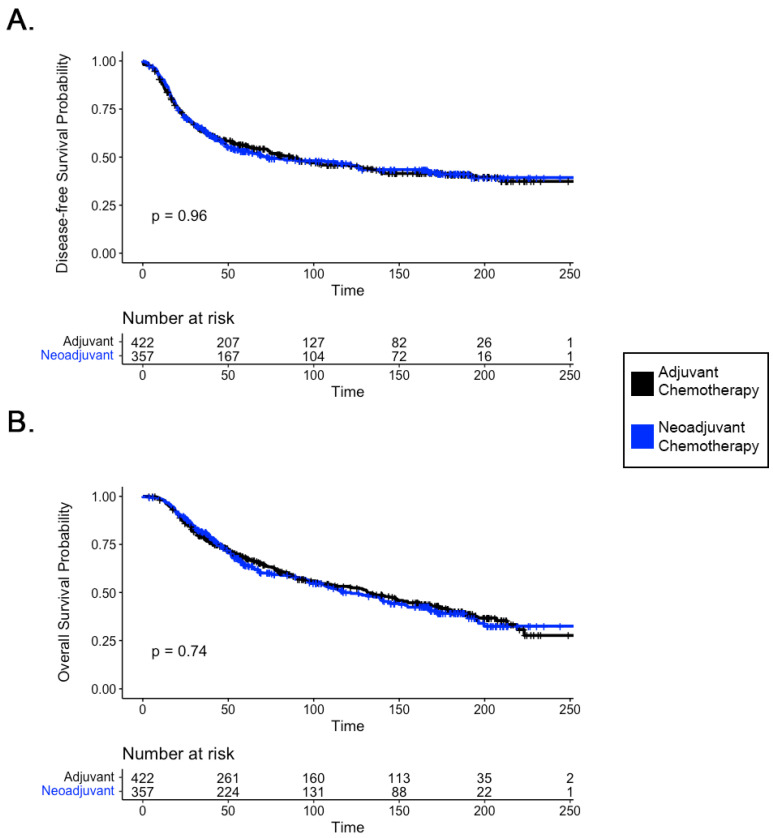
Comparison of neoadjuvant and adjuvant chemotherapy regimens in terms of (**A**) disease-free survival (DFS) and (**B**) overall survival (OS) rates amongst patients with locally advanced breast cancer (LABC) in 6 RCTs identified as sources for the combined RCT patient analysis. No significant differences were found in DFS or OS survival between neoadjuvant versus adjuvant chemotherapeutic regimens.

The DFS rate for neoadjuvant chemotherapy patients collected from all RCTs was 47.90%, whereas patients who received adjuvant treatment had a rate of 47.39%. The OS rate for the neoadjuvant treatment group was 49.58%, while that for the adjuvant treatment group was 49.76%. No significant differences were found with respect to OS (*p* = 0.848) or DFS (*p* = 0.332) amongst patients with LABC receiving neoadjuvant versus adjuvant chemotherapy (Figure 1).

### 3.2. Delay to Surgery

To identify the incidence of any delays to surgery (time from last dose of cytotoxic chemotherapy to operation of >8 weeks) in patients receiving neoadjuvant chemotherapy, our comprehensive, prospectively collected institutional database was analyzed. Our cohort included 563 patients with non-metastatic breast cancer who received preoperative chemotherapy. Of these patients, 164 (29.1%) had early-stage disease, and 399 (70.9%) had LABC. Patient and tumor characteristics were grouped based on delay to surgery and are presented in Table 3. The median time to surgery after neoadjuvant chemotherapy was 32 days (26–41 days interquartile range [IQR], Supplementary Figure S1).

A total of 31 (5.5%) patients had a time interval of over 8 weeks between the last dose of cytotoxic chemotherapy and surgery. The reason for the delay in the 31 patients is included in Table 4. Of these, a total of 13 patients (2.3% of the cohort) had chemotoxicity, the recovery from which mandated the surgical date to be postponed. The planned chemotherapy regimen and associated toxicity in these 13 patients are provided in Supplementary Table S4.

## 4. Discussion

There is a growing trend towards using neoadjuvant chemotherapy in breast cancer treatment [16]. This approach confers several advantages, including the facilitation of breast-conserving surgeries, eradication of axillary metastasis, and allowing in vivo assessments of tumor response to systemic therapies [15,17,18].

The clinical management of locally advanced breast cancer (LABC) differs significantly from that of early-stage disease [19]. LABC is associated with a higher incidence of distant metastasis and poorer survival outcomes compared to early-stage breast cancer [20], highlighting the need to investigate outcomes in this specific patient subset independently. In the early-stage breast cancer population, a large-scale meta-analysis of individual patient data by the Early Breast Cancer Trialists’ Collaborative Group (EBCTCG) found no survival disadvantage to the preoperative use of systemic chemotherapy [9]. The result of our analysis confirms that this finding also extends to patients with LABC receiving neoadjuvant versus adjuvant chemotherapy, demonstrating no significant difference in disease-free and overall survival (Figure 1). 

The time interval between treatment modalities is an important consideration in the treatment of breast cancer. A time interval of over 12 weeks between surgery and adjuvant chemotherapy has been correlated with worse outcomes [21,22]. In the neoadjuvant setting, an interval of less than 8 weeks is considered optimal, as delays beyond this period are associated with worse survival outcomes [11,23,24,25]. This highlights a concern about preoperative chemotherapy; patients may experience complications from chemotherapy, which, if it resulted in a delay to surgery, could, in turn, lead to suboptimal cancer outcomes. 

The data from the aforementioned RCTs lacked the detailed information needed to assess the causes of any delays to surgery. Therefore, local data were used for this purpose. In our cohort, only 2.3% (13 of 563) of patients receiving neoadjuvant chemotherapy experienced a chemotherapy-related delay to surgery (Table 4). Most delays (>8 weeks) were related to administrative reasons such as coordinating reconstructive time with plastic surgery. This underscores the need for better access to the operating room to remedy these potential delays in Canadian healthcare.

Of note, the local cohort in this study only captured patients who underwent surgical intervention. As such, patients with disease progression while on neoadjuvant chemotherapy negating the possibility of surgery were not included in the dataset. Caudle et al. reported on 1928 patients with stage I–III breast cancer undergoing neoadjuvant chemotherapy and found that 0.36% (7 of 1928 patients) had disease progression and did not undergo surgery. Further, only 0.16% (3 of 1928 patients) were candidates for breast-conserving surgery prior to systemic treatment but had to undergo mastectomy due to progression [26]. As such, progression during neoadjuvant chemotherapy interfering with surgical intervention appears to be an infrequent event.

A further limitation of this study is the lack of clinically relevant molecular information, such as HER2 status in the RCTs, due to the period during which these studies were conducted. As noted in the Supplementary Table S2, the chemotherapy regimens in the trials included lacked targeted therapies such as trastuzumab or pertuzumab for HER2-positive disease, and regimens such as 3M (mitomycin C, mitoxantrone, methotrexate) or 2M (mitoxantrone, methotrexate) used in Makris et al. [5] are no longer first-line treatments in breast cancer. Although this impacts the generalizability of our findings to current treatment regimens, it still highlights the lack of difference in outcomes regarding the preoperative vs. postoperative use of chemotherapy in LABC.

Residual disease after neoadjuvant chemotherapy is a strong prognostic indicator in breast cancer [27,28,29]. The data from several clinical trials suggest that neoadjuvant chemotherapy use can identify the subgroup of patients with residual disease who may then benefit from additional adjuvant therapy. Patients with residual disease and breast cancers with a triple-negative subtype in the CREATE-X trial [12] and HER2-positive subtype in the KATHERINE trial [13] were shown to have improved OS and DFS outcomes with added adjuvant regimens. Evidence for additional adjuvant treatment in the hormone receptor-positive HER2-negative cancers with residual disease is lacking as the penelopeB trial did not show improved invasive DFS with the addition of the CDK4/6 inhibitor palbociclib [30]. Molecular analyses may help further guide additional treatment in patients with residual disease after neoadjuvant chemotherapy to optimize outcomes [31].

Risk classification of tumors with molecular signatures such as Oncotype DX^®^ has shown value in the adjuvant setting in identifying patients who would benefit most from systemic therapy and de-escalating treatment for those with low-risk disease [32]. A potential drawback of administering the chemotherapy preoperatively, therefore, could be the overtreatment of patients with low-risk disease [33]. Gene assays of the biopsy specimen have the potential to help guide neoadjuvant treatment for patients with hormone receptor-positive cancers [34]. Therefore, concerns about overtreatment with the neoadjuvant approach could be potentially avoided by using molecular signatures of the tumor phenotype as opposed to only the clinical information to guide treatment decisions. The trials included in our study were conducted prior to standard testing for HER2 and, therefore, lacked detailed molecular classification of tumors. While it is unlikely that randomized trials of this nature will ever be repeated, it is possible that future analyses of neoadjuvant treatment with molecular subsets of patients at the highest likelihood to benefit from neoadjuvant chemotherapy may reveal a survival advantage in comparison to adjuvant systemic therapy. Nevertheless, the absence of significant downsides to the neoadjuvant chemotherapy approach for patients for whom chemotherapy is known to have a survival benefit should favor its use in order to maximize the therapeutic options for patients with residual disease. Additionally, the preliminary results of the NSABP B-51 trial have shown that patients who have had axillary nodal down-staging after neoadjuvant chemotherapy may safely avoid regional nodal irradiation, providing another support for the preoperative use of chemotherapy [35]. Patients with nodal positivity can be surgically down-staged from axillary dissection to sentinel node biopsy in patients who respond to neoadjuvant chemotherapy, avoiding the surgical morbidity of lymphedema and chronic pain associated with axillary dissection surgery [36,37,38].

Our study demonstrates that neoadjuvant chemotherapy has similar survival outcomes in the LABC cohort as those receiving adjuvant systemic treatment. Delays to surgery because of chemotherapy complications are rare, and this concern should not impact patient access to neoadjuvant treatments. Evolving data support additional adjuvant therapy in a select group of patients with residual disease after neoadjuvant therapy, suggesting a potential survival advantage to this approach. This study further supports the paradigm shift towards the preoperative use of systemic therapy in the treatment of breast cancer.

## Figures and Tables

**Table 1:** Baseline characteristics of LABC patients enrolled in all six identified RCTs used in the analysis.

RCT	Mean Age, Years (SD)	ER Status		PR Status		Surgery Type		
		− (freq)	+ (freq)	− (freq)	+ (freq)	Lumpectomy/ Conservative	Mastectomy	Biopsy/ No Surgery
Wolmark et al. [15]	48.9 (11.1)	166	182	186	161	139	287	2
Deo et al. [3]	46.6 (10.6)	77	102	79	100	0	179	0
Gazet et al. [4]	54.8 (8.8)	30	0	0	0	12	12	2
Makris et al. [5]	47.7 (6.1)	4	3	0	0	2	5	0
Mauriac et al. [6]	52.8 (5.5)	36	20	38	18	8	40	8
Van der Hage et al. [7]	48.6 (9.8)	0	0	0	0	3	76	12

ER, estrogen receptor; PR, progesterone receptor. Frequency was missing for 197 patients.

**Table 2:** Clinical nodal status and N stage of patients enrolled in all six identified RCTs included in the statistical analysis.

RCT	Clinical Nodal Status	Pathological N Stage, Number of Positive Nodes				Total
	Negative (N0)	0	1–3	4–9	>10	
Wolmark et al. [15]	232	98	82	158	86	424
Deo et al. [3]	0	51	46	38	44	179
Gazet et al. [4]	0	21	8	1	0	30
Makris et al. [5]	0	4	1	2	0	7
Mauriac et al. [6]	27	15	19	9	5	48
Van der Hage et al. [7]	0	10	25	24	19	78

Pathologic N stage data are missing for 13 patients; clinical nodal status is missing for 295 patients.

**Table 3:** Clinical characteristics of local cohort of breast cancer patients with non-metastatic breast cancer undergoing neoadjuvant chemotherapy. Of the 563 patients, 31 (5.5%) experienced a delay of over 8 weeks to surgery.

Characteristic	Overall, N = 563 ^1^	Time to Surgery		*p*-Value
		>8 Weeks, N = 31 (5.5%) ^1^	≤8 Weeks, N = 532 (94.5%) ^1^	
Age	51 (43, 61)	62 (52, 67)	50 (42, 60)	<0.001
cT stage				0.3
T1	39 (6.9%)	1 (3.2%)	38 (7.1%)	
T2	155 (28%)	7 (23%)	148 (28%)	
T3	204 (36%)	9 (29%)	195 (37%)	
T4	165 (29%)	14 (45%)	151 (28%)	
cN stage				0.3
N0	224 (40%)	9 (29%)	215 (40%)	
N1	274 (49%)	16 (52%)	258 (48%)	
N2	45 (8.0%)	5 (16%)	40 (7.5%)	
N3	20 (3.6%)	1 (3.2%)	19 (3.6%)	
ER status				>0.9
Negative	203 (36%)	11 (35%)	192 (36%)	
Positive	360 (64%)	20 (65%)	340 (64%)	
PR status				0.079
Negative	259 (46%)	19 (61%)	240 (45%)	
Positive	304 (54%)	12 (39%)	292 (55%)	
Her2 status				0.5
Negative	350 (62%)	21 (68%)	329 (62%)	
Positive	213 (38%)	10 (32%)	203 (38%)	
Grade				>0.9
1	35 (6.2%)	1 (3.2%)	34 (6.4%)	
2	240 (43%)	14 (45%)	226 (42%)	
3	288 (51%)	16 (52%)	272 (51%)	
Subtype				0.8
Her2	75 (13%)	4 (13%)	71 (13%)	
Lum_A	151 (27%)	10 (32%)	141 (27%)	
Lum_B	217 (39%)	10 (32%)	207 (39%)	
TN	120 (21%)	7 (23%)	113 (21%)	
Time to Surgery (days)	32 (26, 41)	79 (63, 115)	32 (25, 39)	

^1^ Median (IQR); n (%).

**Table 4:** Thirty-one patients experienced a time gap of over 8 weeks from the last dose of cytotoxic chemotherapy to surgery. Of those delays, 13 were because of an adverse reaction to chemotherapy. Overall, chemotoxicity causing a delay to surgery represents 2.3% (13 of 563) of patients undergoing neoadjuvant chemotherapy.

Characteristic	N = 31 ^1^
Reason_for_delay	
Administrative/Non-Medical	9 (29%)
Chemotoxicity	13 (42%)
Clinical Trial	7 (23%)
Medical reasons unrelated to chemotherapy	2 (6%)

^1^ n (%).

## Data Availability

The datasets presented in this article are not readily available due to institutional restrictions concerning patient privacy.

## Supplementary Materials

The following supporting information can be downloaded at: https://www.mdpi.com/article/10.3390/curroncol31100448/s1, Table S1: Breast cancer subtype assignment in the local cohort based on ER, PR, Her2 status and tumour grade; Table S2: Comparison of chemotherapy regimen and number of patients enrolled in all six identified RCTs included in the meta-analysis; Table S3: Statistical comparison (*p*-values) of derived HR of DFS and OS rate between neoadjuvant and adjuvant chemotherapy regimens of patients with LABC in all six RCTs identified as sources for the combined statistical analysis. Table S4: Chemotherapy regimens in local cohort of patients experiencing delay to surgery (n = 13), and the associated reaction. Figure S1: Histogram illustrating distribution of time since the last dose of cytotoxic chemotherapy to surgery in the local cohort. Red line denotes 8 weeks (56 days).

## References

[1] Ikeda T., Jinno H., Matsui A., Masamura S., Kitajima M. (2002). The Role of Neoadjuvant Chemotherapy for Breast Cancer Treatment. Breast Cancer.

[2] Fisher B., Brown A., Mamounas E., Wieand S., Robidoux A., Margolese R.G., Cruz A.B., Fisher E.R., Wickerham D.L., Wolmark N. (1997). Effect of Preoperative Chemotherapy on Local-Regional Disease in Women with Operable Breast Cancer: Findings from National Surgical Adjuvant Breast and Bowel Project B-18. J. Clin. Oncol..

[3] Deo S.V.S., Bhutani M., Shukla N.K., Raina V., Rath G.K., Purkayasth J. (2003). Randomized Trial Comparing Neo-adjuvant versus Adjuvant Chemotherapy in Operable Locally Advanced Breast Cancer (T4b N0-2 M0). J. Surg. Oncol..

[4] Gazet J.-C., Ford H.T., Gray R., McConkey C., Sutcliffe R., Quilliam J., Makinde V., Lowndes S., Coombes R.C. (2001). Estrogen-Receptor-Directed Neoadjuvant Therapy for Breast Cancer: Results of a Randomised Trial Using Formestane and Methotrexate, Mitozantrone and Mitomycin C (MMM) Chemotherapy. Ann. Oncol..

[5] Makris A., Powles T.J., Ashley S.E., Chang J., Hickish T., Tidy V.A., Nash A.G., Ford H.T. (1998). A Reduction in the Requirements for Mastectomy in a Randomized Trial of Neoadjuvant Chemoendocrine Therapy in Primary Breast Cancer. Ann. Oncol..

[6] Mauriac L., MacGrogan G., Avril A., Durand M., Floquet A., Debled M., Dilhuydy J.M., Bonichon F., (IBBGS) I.B.B.G.S. (1999). Neoadjuvant Chemotherapy for Operable Breast Carcinoma Larger than 3 cm: A Unicentre Randomized Trial with a 124-Month Median Follow-Up. Ann. Oncol..

[7] van der Hage J.A., van de Velde C.J., Julien J.P., Tubiana-Hulin M., Vandervelden C., Duchateau L. (2001). Preoperative Chemotherapy in Primary Operable Breast Cancer: Results from the European Organization for Research and Treatment of Cancer Trial 10902. J. Clin. Oncol..

[8] Brackstone M., Fletcher G.G., Dayes I.S., Madarnas Y., SenGupta S.K., Verma S., Members of the Breast Cancer Disease Site Group (2015). Locoregional Therapy of Locally Advanced Breast Cancer: A Clinical Practice Guideline. Curr. Oncol..

[9] (EBCTCG) E.B.C.T.C.G., Asselain B., Barlow W., Bartlett J., Bergh J., Bergsten-Nordström E., Bliss J., Boccardo F., Boddington C., Bogaerts J. (2018). Long-Term Outcomes for Neoadjuvant versus Adjuvant Chemotherapy in Early Breast Cancer: Meta-Analysis of Individual Patient Data from Ten Randomised Trials. Lancet Oncol..

[10] Bleicher R.J. (2018). Timing and Delays in Breast Cancer Evaluation and Treatment. Ann. Surg. Oncol..

[11] Cullinane C., Shrestha A., Maksoud A.A., Rothwell J., Evoy D., Geraghty J., McCartan D., McDermott E.W., Prichard R.S. (2021). Optimal Timing of Surgery Following Breast Cancer Neoadjuvant Chemotherapy: A Systematic Review and Meta-Analysis. Eur. J. Surg. Oncol..

[12] Norikazu M., Soo-Jung L., Shoichiro O., Young-Hyuck I., Eun-Sook L., Isao Y., Katsumasa K., Seock-Ah I., Byeong-Woo P., Sung-Bae K. (2017). Adjuvant Capecitabine for Breast Cancer after Preoperative Chemotherapy. N. Engl. J. Med..

[13] Von Minckwitz G., Huang C.-S., Mano M.S., Loibl S., Mamounas E.P., Untch M., Wolmark N., Rastogi P., Schneeweiss A., Redondo A. (2018). Trastuzumab Emtansine for Residual Invasive HER2-Positive Breast Cancer. N. Engl. J. Med..

[14] Amin M.B., Greene F.L., Edge S.B., Compton C.C., Gershenwald J.E., Brookland R.K., Meyer L., Gress D.M., Byrd D.R., Winchester D.P. (2017). The Eighth Edition AJCC Cancer Staging Manual: Continuing to Build a Bridge from a Population-based to a More “Personalized” Approach to Cancer Staging. CA Cancer J. Clin..

[15] Wolmark N., Wang J., Mamounas E., Bryant J., Fisher B. (2001). Preoperative Chemotherapy in Patients With Operable Breast Cancer: Nine-Year Results From National Surgical Adjuvant Breast and Bowel Project B-18. JNCI Monogr..

[16] Graham P.J., Brar M.S., Foster T., McCall M., Bouchard-Fortier A., Temple W., Quan M.L. (2015). Neoadjuvant Chemotherapy for Breast Cancer, Is Practice Changing? A Population-Based Review of Current Surgical Trends. Ann. Surg. Oncol..

[17] Chen Y., Shi X.-E., Tian J.-H., Yang X.-J., Wang Y.-F., Yang K.-H. (2018). Survival Benefit of Neoadjuvant Chemotherapy for Resectable Breast Cancer. Medicine.

[18] Buchholz T.A., Hunt K.K., Whitman G.J., Sahin A.A., Hortobagyi G.N. (2003). Neoadjuvant Chemotherapy for Breast Carcinoma. Cancer.

[19] Aebi S., Karlsson P., Wapnir I.L. (2022). Locally Advanced Breast Cancer. Breast.

[20] Nordenskjöld A.E., Helena F., Arnesson L.G., Einbeigi Z., Holmberg E., Albertsson P., Karlsson P. (2019). Breast Cancer Survival Trends in Different Stages and Age Groups—A Population-Based Study 1989–2013. Acta Oncol..

[21] Cai L., Tong Y., Zhu X., Shen K., Zhu J., Chen X. (2020). Prolonged Time to Adjuvant Chemotherapy Initiation Was Associated with Worse Disease Outcome in Triple Negative Breast Cancer Patients. Sci. Rep..

[22] Kupstas A.R., Hoskin T.L., Day C.N., Habermann E.B., Boughey J.C. (2019). Effect of Surgery Type on Time to Adjuvant Chemotherapy and Impact of Delay on Breast Cancer Survival: A National Cancer Database Analysis. Ann. Surg. Oncol..

[23] Suleman K., Almalik O., Haque E., Mushtaq A., Badran A., Alsayed A., Ajarim D., Al-Tweigeri T., Jastaniyah N., Elhassan T. (2020). Does the Timing of Surgery after Neoadjuvant Therapy in Breast Cancer Patients Affect the Outcome?. Oncology.

[24] Sanford R.A., Lei X., Barcenas C.H., Mittendorf E.A., Caudle A.S., Valero V., Tripathy D., Giordano S.H., Chavez-MacGregor M. (2016). Impact of Time from Completion of Neoadjuvant Chemotherapy to Surgery on Survival Outcomes in Breast Cancer Patients. Ann. Surg. Oncol..

[25] Al-Masri M., Aljalabneh B., Al-Najjar H., Al-Shamaileh T. (2021). Effect of Time to Breast Cancer Surgery after Neoadjuvant Chemotherapy on Survival Outcomes. Breast Cancer Res. Treat..

[26] Caudle A.S., Gonzalez-Angulo A.M., Hunt K.K., Pusztai L., Kuerer H.M., Mittendorf E.A., Hortobagyi G.N., Meric-Bernstam F. (2011). Impact of Progression During Neoadjuvant Chemotherapy on Surgical Management of Breast Cancer. Ann. Surg. Oncol..

[27] Kuerer H.M., Newman L.A., Smith T.L., Ames F.C., Hunt K.K., Dhingra K., Theriault R.L., Singh G., Binkley S.M., Sneige N. (1999). Clinical Course of Breast Cancer Patients with Complete Pathologic Primary Tumor and Axillary Lymph Node Response to Doxorubicin-Based Neoadjuvant Chemotherapy. J. Clin. Oncol..

[28] Chollet P., Amat S., Cure H., de Latour M., Bouedec G.L., Mouret-Reynier M.-A., Ferriere J.-P., Achard J.-L., Dauplat J., Penault-Llorca F. (2002). Prognostic Significance of a Complete Pathological Response after Induction Chemotherapy in Operable Breast Cancer. Br. J. Cancer.

[29] Amat S., Abrial S.C., Abrial C., Penault-Llorca F., Delva R., Bougnoux P., Leduc B., Mouret-Reynier M.-A., Mery-Mignard D., Bleuse J.-P. (2005). High Prognostic Significance of Residual Disease after Neoadjuvant Chemotherapy: A Retrospective Study in 710 Patients with Operable Breast Cancer. Breast Cancer Res. Treat..

[30] Loibl S., Marmé F., Martin M., Untch M., Bonnefoi H., Kim S.-B., Bear H., McCarthy N., Olivé M.M., Gelmon K. (2021). Palbociclib for Residual High-Risk Invasive HR-Positive and HER2-Negative Early Breast Cancer—The Penelope-B Trial. J. Clin. Oncol..

[31] Foldi J., Rozenblit M., Park T.S., Knowlton C.A., Golshan M., Moran M., Pusztai L. (2021). Optimal Management for Residual Disease Following Neoadjuvant Systemic Therapy. Curr. Treat. Options Oncol..

[32] Sparano J.A., Gray R.J., Makower D.F., Pritchard K.I., Albain K.S., Hayes D.F., Geyer C.E., Dees E.C., Goetz M.P., Olson J.A. (2018). Adjuvant Chemotherapy Guided by a 21-Gene Expression Assay in Breast Cancer. N. Engl. J. Med..

[33] Kantor O., Barrera E., Kopkash K., Pesce C., Barrera E., Winchester D.J., Yao K. (2019). Are We Overtreating Hormone Receptor Positive Breast Cancer with Neoadjuvant Chemotherapy? Role of OncotypeDx^®^ for Hormone Receptor Positive Patients Undergoing Neoadjuvant Chemotherapy. Ann. Surg. Oncol..

[34] Bear H.D., Wan W., Robidoux A., Rubin P., Limentani S., White R.L., Granfortuna J., Hopkins J.O., Oldham D., Rodriguez A. (2017). Using the 21-gene Assay from Core Needle Biopsies to Choose Neoadjuvant Therapy for Breast Cancer: A Multicenter Trial. J. Surg. Oncol..

[35] Mamounas E.P., Bandos H., White J.R., Julian T.B., Khan A.J., Shaitelman S.F., Torres M.A., Vicini F., Ganz P.A., McCloskey S.A. (2019). NRG Oncology/NSABP B-51/RTOG 1304: Phase III Trial to Determine If Chest Wall and Regional Nodal Radiotherapy (CWRNRT) Post Mastectomy (Mx) or the Addition of RNRT to Whole Breast RT Post Breast-Conserving Surgery (BCS) Reduces Invasive Breast Cancer Recurrence-Free Interval (IBCR-FI) in Patients (Pts) with Pathologically Positive Axillary (PPAx) Nodes Who Are YpN0 after Neoadjuvant Chemotherapy (NC). J. Clin. Oncol..

[36] Boughey J.C., Suman V.J., Mittendorf E.A., Ahrendt G.M., Wilke L.G., Taback B., Leitch A.M., Kuerer H.M., Bowling M., Flippo-Morton T.S. (2013). Sentinel Lymph Node Surgery after Neoadjuvant Chemotherapy in Patients with Node-Positive Breast Cancer: The ACOSOG Z1071 (Alliance) Clinical Trial. JAMA.

[37] Kuehn T., Bauerfeind I., Fehm T., Fleige B., Hausschild M., Helms G., Lebeau A., Liedtke C., von Minckwitz G., Nekljudova V. (2013). Sentinel-Lymph-Node Biopsy in Patients with Breast Cancer before and after Neoadjuvant Chemotherapy (SENTINA): A Prospective, Multicentre Cohort Study. Lancet Oncol..

[38] Boileau J.-F., Poirier B., Basik M., Holloway C.M.B., Gaboury L., Sideris L., Meterissian S., Arnaout A., Brackstone M., McCready D.R. (2014). Sentinel Node Biopsy After Neoadjuvant Chemotherapy in Biopsy-Proven Node-Positive Breast Cancer: The SN FNAC Study. J. Clin. Oncol..

