# Supplementary material for: The Impact of Neoadjuvant versus Adjuvant Chemotherapy on Survival Outcomes in Locally Advanced Breast Cancer
Source: Curr Oncol. 2024 Oct 8;31(10):6007–16. doi: 10.3390/curroncol31100448 (PMC11505903; doi:10.3390/curroncol31100448)
Supplement: Supplementary file 1 [file curroncol-31-00448-s001.zip › curroncol-3203409-supplementary.pdf]

SUPPLEMENTARY DATA

Supplementary Table S1. Breast cancer subtype assignment in the local cohort based on ER, PR, Her2 status and tumour grade.

Subtype	ER/PR-status	Her2-status	Tumour Grade
**Triple Negative (TN)**	Negative	Negative	Any
**Her2-positive (Her2)**	Negative	Positive	Any
**Luminal A (Lum A)**	Positive	Negative	1 and 2
**Luminal B (Lum B)**	Positive	Positive	Any
	Positive	Negative	3

Supplementary Table S2. Comparison of chemotherapy regimen and number of patients enrolled in all six identified RCTs included in the meta-analysis. (*AC*, adriamycin-cyclophosphamide; *CAF*, cyclophosphamide-adriamycin-fluorouracil; *CEF*, cyclophosphamide-epirubicin-fluorouracil; *CMF*, cyclophosphamide-methotrexate-fluorouracil; *MMM*, mitomycine-mitozantrone-methotrexate; *MM*, mitozantrone-methotrexate.).

	Study dates	Chemotherapy Regimen	Patient Enrollment		Patients Stratified into Therapy	
					LABC Patients, N (%)	
			All, N	LABC, N (%Total)	Neoadjuvant	Adjuvant
**Wolmark *et al.*** 2001	1998-1993	AC	918	428/918 (46.7%)	230/428 (53.7%)	198/428 (46.3%)
**Deo *et al.*** 2003	1997-2001	CAF, CAF/CEF, CEF/ CEF/CAF, CMF/CAF	179	179/179 (100%)	89/179 (49.7%)	90/179 (50.3%)
**Gazet *et al.*** 2001	1990-1993	3M (methotrexate, mitozantrone, and mitomycin)	108	30/108 (27.8%)	15/30 (50.0%)	15/30 (50.0%)
**Makris *et al.*** 1998	1990-1995	mitomycin C, mitoxantrone, methotrexate (3M) or mitoxantrone, methotrexate (2M)	165	7/165 (4.2%)	3/7 (42.9%)	4/7 (57.1%)
**Mauriac *et al.*** 1999	1985-1989	epirubicin , vincristine, methotrexate then mitomycin C, thiotepa, vindesine	238	56/238 (23.6%)	33/56 (58.9%)	23/56 (41.1%)
**Van der Hage *et al.*** 2001	1991-1999	FEC	698	79/698 (11.3%)	52/79 (65.8%)	27/79 (34.2%)
**Total**			**2306**	**779/2306 (33.8%)**	**422**	**357**

Supplementary Table S3. Statistical comparison (p-values) of derived HR of DFS and OS rate between neoadjuvant and adjuvant chemotherapy regimens of patients with LABC in all six RCTs identified as sources for the combined statistical analysis.

RCT	p-value	
	DFS	OS
**Wolmark et al.**[15]	0.733	0.952
**Deo et al.** [3]	0.235	0.468
**Gazet et al.** [4]	0.156	0.786
**Makris et al.** [5]	0.540	0.540
**Mauriac et al.** [6]	0.639	0.478
**Van der Hage et al.** [7]	0.202	0.515
**Combined-Adjusted for Trial**	**0.891**	**0.891**

Supplementary Table S4. Chemotherapy regimens in local cohort of patients experiencing delay to surgery (n=13), and the associated reaction.

Planned Regimen	Reaction	Number of patients
**AC-T**	Pneumonitis	1
	Peripheral Neuropathy	1
	Infection and neutropenia	4
	Infusion reaction to paclitaxel	2
**AC-T + Trastuzumab**	Cardiotoxicity with Trastuzumab	2
**FEC-D**	Infection and neutropenia	1
**Paclitaxel**	Progressive shortness of breath	1
**CMF + Trastuzumab**	Infection and neutropenia	1

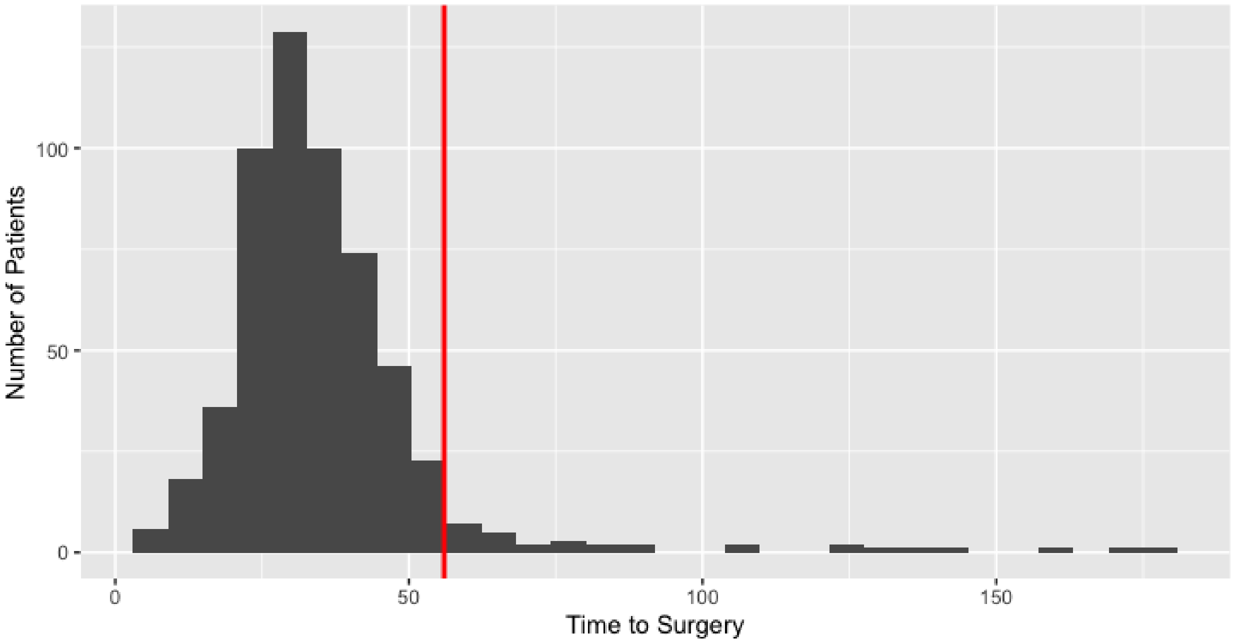

Supplementary Figure S1. Histogram illustrating distribution of time since the last dose of cytotoxic chemotherapy to surgery in the local cohort. Red line denotes 8 weeks (56 days).
